# Application of the Esscher Transform to Pricing Forward Contracts on Energy Markets in a Fuzzy Environment

**DOI:** 10.3390/e25030527

**Published:** 2023-03-18

**Authors:** Piotr Nowak, Michał Pawłowski

**Affiliations:** Systems Research Institute, Polish Academy of Sciences, Newelska 6, 01-447 Warsaw, Poland

**Keywords:** energy markets, fuzzy sets, stochastic processes, jump-diffusion, derivatives pricing, decision making

## Abstract

The paper is dedicated to modeling electricity spot prices and pricing forward contracts on energy markets. The underlying dynamics of electricity spot prices is governed by a stochastic mean reverting diffusion with jumps having mixed-exponential distribution. Application of financial mathematics and stochastic methods enabled the derivation of the analytical formula for the forward contract’s price in a crisp case. Since the model parameters’ incertitude is considered, their fuzzy counterparts are introduced. Utilization of fuzzy arithmetic enabled deriving an analytical expression for the futures price and proposing a modified method for decision-making under uncertainty. Finally, numerical examples are analyzed to illustrate our pricing approach and the proposed financial decision-making method.

## 1. Introduction

For almost three decades, the European electricity market has undergone gradual transition. In the early 1990s, the energy sector was monopolized with vertically integrated enterprises engaged in production, transportation, supply and holding of the grid infrastructure. Electricity prices were therefore dictated by these companies, in the absence of competition. In 1996, however, the European Union set out to liberalize and integrate the internal energy market with a key step to unbundle the power sector, in order to reduce the whole transportation costs, increase the security of supply and to enable access to the market for new entities. The second directive from 2003 imposed, i.a., the possibility to choose the energy supplier by all European customers by 2007. The turn of the millennium was also a period of the emergence of energy exchanges in Europe.

Electricity spot day-ahead prices exhibit distinguishing traits such as seasonality (the consequence of substantial variation of demand throughout the year and week), huge volatility and sudden spikes (due to transmission failures, generation outages, weather changes, etc., in combination with inelasticity of production and consumption of energy and infeasibility of storage), mean-reversion (fluctuation around the marginal cost of generation, the price is forced back to its long-run mean after a jump as soon as additional producers enter the market). Existence of spikes do not affect forward prices, inasmuch as they are short-lived.

The 1973 Black–Scholes approach, groundbreaking for financial mathematics, enabled derivation of an analytical formula for pricing European options, using geometric Brownian motion as the underlying asset price process. However, the shortcomings of the Black–Scholes model and proposed alternative solutions to the option pricing problem have been known for many years. Since the literature concerning this issue is very rich, we mention only several articles. Among others, Levy processes, including mixed-exponential jump-diffusion (MEM) in [1] and the models in [2,3], were proposed to describe the underlying assets’ log prices. The papers [4,5] were devoted to modeling the underlying assets with application of Levy jump-diffusions under uncertainty, involving semimartingale characteristics (see, e.g., [6]) for the options valuation. Other models based on the processes with independent and stationary increments were presented in [7], taking the form of the Markov-modulated Levy processes, as well as in the paper [8], where the Markovian regime switching exponential Levy process was applied together with interest rates modeled by the Markovian regime switching Hull–White process. In turn, ref. [9] used a process with pure jumps. The Vasicek stochastic interest rate and a general Levy process of an underlying asset were considered in [10], including the jump-diffusion and the infinite activities Levy process as special cases. It is worth mentioning that diffusions and jump-diffusions were also used for other financial instruments with complex payoff structures (see, e.g., ref. [11] for the case of catastrophe bond pricing).

Market prices of financial instruments fluctuate, which often makes it impossible to determine the model parameters values precisely. Therefore, the knowledge of experts replaces statistical techniques. This replacement is conducted by employing imprecise information models, most often in the form of fuzzy numbers. This article falls within the trend of valuation methods that combine stochastic modeling with the application of fuzzy set theory. However, unlike most of them, which are devoted to options pricing, it deals with the valuation of forward contracts on the energy exchange. Essential extensions—compared to our previous paper [12]—are introducing a more general form of jump distribution, which enables the possibility of negative jumps, and a modified decision-making method. Similarly, as for other stochastic models of primary financial instruments, a small model change causes a significant challenge to valuing the derivative instrument, even in the crisp form. That was also the case for the model proposed in this paper. The process of derivation of forward contracts prices was not just a simple transfer of the approach from the [12]. It involved advanced stochastic analysis methods due to the change of the probability measure to an equivalent one, using the Esscher transform. In particular, the change in the spot price model complicated the conditions that should be satisfied to make the mentioned equivalent change of measure feasible. It also significantly impacted the form of the fuzzy analytical formula for forward contracts pricing. In turn, the proposed modification of the financial decision-making method is of practical importance. The section with numerical examples includes the comparison of the three investment decision-making methods: the primary one proposed in [13,14], the reduced one tested in [12] and the modified, current method, highlighting the limitations of the former. Apart from removing the inconclusive set of investment recommendations (combining accumulate, hold and reduce), potentially possible to occur in the method from [13,14], and the empty set of recommendations (feasible in the method presented in [12]), the present method makes use of the weighted possibilistic mean, which is a single numeric representation of elements of a fuzzy number. This notion, used adequately, influences the outcomes of the procedure, i.a., makes them more unambiguous for some combination of the model and market variables.

Another advantage of the new method refers to the weighted possibilistic mean itself—the weighting function allows to put emphasis on a subset of the support of a fuzzy number on which the function achieves bigger values. If this function is increasing, bigger weights are assigned to elements closer to the modal value.

Recently, other types of fuzzy sets have also been adapted for decision making. Ref. [15] has proposed an efficient algorithm for interval-valued fuzzy soft sets, which involves less computation than the existing ones. A new interval-valued intuitionistic fuzzy soft sets-based approach to decision making and three parameter reduction algorithms have been presented in [16]. The proposed method, based on choice value and score value of membership/nonmembership degrees, helps decision-makers choose a unique option.

We present a mean-reverting jump-diffusion process of dynamics of electricity day-ahead spot prices. The explained above typical patterns observed in electricity prices time series are reproduced within the model. Apart from that, it permits adjustment to the commonly noticed inverse leverage effect (see, e.g., [17]). Namely, the marginal production cost is a convex and increasing function of production volume. As a result, an increase in load causes a higher proportional increase in equilibrium price which in turn entails asymmetry in volatility. This excess variability “stretches out” the right tail of the spot price log-returns distribution causing the positive skewness and heavier right tail (contrary, e.g., to equity markets where the left tail is fatter) compared to the log-normal distribution (assumed in the Black–Scholes approach). Existence of positive spikes and negative jumps of prices (part of which is seasonal, occurring, e.g., on holidays when the load decreases, and thus deterministic) contributes to leptokurticism. The parameters describing the mixed-exponential distribution of jumps and the appropriate form of the seasonality function allow to adjust both the skewness and kurtosis of the distribution of the spot price log-returns.

Introduction of the fuzzy approach to pricing of derivatives aims at investigating the influence of incertitude of values of model parameters on a price of a derivative. This uncertainty arises from volatility of variables observed in the market and from imprecision of estimation of unobserved model parameters. The presented fuzzy approach allows to obtain prices of derivatives as fuzzy numbers. However, the form of the resulting derivative’s fuzzy price depends on the type of fuzzy numbers chosen for parameters and on the choice of the pricing method in the crisp case.

The contribution of the paper is fourfold. (i) We introduce a stochastic mean reverting diffusion with jumps having the mixed-exponential distribution to model spot prices on energy markets. This jump distribution enables positive and negative jumps. It also can approximate many probability distributions used in financial modeling. (ii) The analytical formula for the forward price, derived in the paper, can be applied for the valuation of forward contracts in the crisp case. We obtain this forward price formula using the Esscher transformed equivalent probability measure and advanced stochastic techniques. (iii) We also derive the analytical fuzzy forward contract’s price in an analytical form. (iv) Finally, we propose a modified financial decision-making method, employing the fuzzy pricing formulas mentioned above. Numerical examples illustrate the theoretical results. In particular, we present an example of application of the decision-making method and conduct the sensitivity analysis of the fuzzy forward contract’s price with respect to the fuzzified volatility.

It is also worth underlining the relationship between Esscher transformed martingale measures and the minimal entropy martingale measure. It was proved in [18] that in the case of geometric Levy processes, the simple return Esscher transformed martingale measure is the minimal entropy martingale measure. Thus, the Esscher transform used in our paper is related to the widely understood notion of entropy.

The paper is organized as follows. Section 2 presents the state-of-the-art, describing some existing electricity crisp prices models and the approaches used to pricing options in a fuzzy environment. The proposed electricity spot prices model is introduced in Section 3. The crisp analytical formula for the forward price is derived in Section 4. Section 5 and Section 6 are devoted to the derivation of the fuzzy pricing formula and description of the financial decision-making method, respectively. Section 7 is dedicated to numerical examples. The final section contains the conclusions.

## 2. Overview of Valuation Methods

### 2.1. Electricity Crisp Prices Models

#### 2.1.1. Jump-Diffusion Models

A prototype one-factor model, which became a milestone in electricity derivatives pricing, is introduced in [19] where the authors take into account seasonality and mean-reversion, however jumps shaping the excess kurtosis and skewness are excluded from consideration. This inadvertence has been eliminated in [20,21] where normally and double exponentially distributed jumps, respectively, are added to the mean-reverting diffusion. There is a possibility of deriving the analytical formula for a forward price within these aforementioned models.

More complex form of the jump process is proposed in [22] where the jump size has a truncated exponential distribution (prevents the occurrence of large price jumps), sign of a jump is dependent on the spread between the value of a seasonality function and actual price (guarantees that two subsequent prices cannot be above a threshold), stochastic intensity of the Poisson process is a product of a function of periodic intensity shape and a function which starts to increase when electricity price exceeds some predefined level indicating the beginning of excess frequency of jumps.

#### 2.1.2. Regime Switching Models

Some electricity markets have tendency to remain in a state of extreme prices for some longer period (in case of day-ahead prices modeling for a period longer than one day)—when, e.g., the system repair prolongs. Then, regime switching models may be more adequate than mean-reverting jump-diffusions inasmuch as they do not force the price to revert immediately to the moderate levels. Instead, one deals with separate regimes for different market conditions, usually downward jump (or negative prices) regime, upward jump regime and base regime with their individual dynamics linked by a transition matrix with probabilities of shifts from one regime to another (see, e.g., [23,24,25]) for sample models.

#### 2.1.3. ARMA Models

The deseasonalized part of the electricity day-ahead price process is often modeled by the autoregressive moving average processes. Nevertheless, due to jumps in prices and volatility clustering, residuals of the process do not fulfill the assumption of being normal, identically distributed and independent. This is why generalized autoregressive conditionally heteroscedastic (GARCH) models are usually assumed to model variance of electricity time series.

#### 2.1.4. Other Approaches

An uncoupling of the jump addend from the mean-reverting diffusion allows to dissociate the mean-reversion coexisting with the diffusion-generated noise from the jump process. Nevertheless, in this case there must exist a separate mechanism making the price process revert to some seasonal level after the abrupt jump. Ref. [26] propose to decompose the jump process to the sum of two processes modeling the positive and negative jumps sampled from a normal distribution. Stochastic jump intensities modeled by mean-reverting jump processes are introduced. As a result, in case of a jump in a spot price, the value of the respective intensity also rapidly grows, making the probability of a reverting jump in the spot price much higher.

A very general class of dynamics of deseasonalized electricity prices may be described by a mean-reverting model with a Levy process as the driving noise. Ref. [27] impose the normal inverse Gaussian distribution (NIG) for the Levy increments and claim a very good fit to spot prices series (the NIG distribution catch heavy tails and a center of the distribution fairly well). They compose a set of equivalent martingale measures by the Esscher transform and find the market price of risk, which minimizes the distance between theoretical and observed forward prices. Due to knowing the characteristic function of the deseasonalized price, it is possible to price options on the spot by using the fast Fourier transform technique.

The idea of the factor model, cf. [28], is a split of an electricity spot price into base and spike signals with dynamics dedicated to any type of prices fluctuations. It allows to include into the model different mean-reversion’s speeds. Namely, the deseasonalized price process is modeled as a weighted sum of independent non-Gaussian Ornstein–Uhlenbeck processes (additive cadlag processes with increasing paths). The extensive analysis of the model has been performed in [29,30].

### 2.2. Fuzzy Approaches to Pricing Derivatives

The issue of option pricing under uncertainty has recently become an important problem addressed by many authors.

In the beginning, it is worth recalling the work [31], which gave rise to the pricing of European options by combining stochastic and fuzzy methods, where the underlying financial instrument was described by a geometric Brownian motion, as in the case of the traditional Black–Scholes model. Within the same continuous model of the underlying instrument, refs. [32,33,34,35] remained, using fuzzy estimation, adaptive fuzzy numbers, or focusing the attention on reload and Asian options. Ref. [36], also applying the geometric Brownian motion, proposed a different approach, based on the rational expected option price depending on a fuzzy goal. Ref. [37] used triangular approximations to improve the fuzzy Black–Scholes option pricing formula.

Refs. [5,13,14,38,39] proposed a generalization of the techniques from [31], introducing the possibility of jumps of the underlying asset prices with application of a Levy jump-diffusion. A similar model was considered in [40], whereas [41] used an infinite pure jump Levy process to model the primary financial instrument. Levy process approaches were also used in [42] for n-fold compound option pricing and in [43] to the valuation of the total return swap.

New complex methods of fuzzy mathematics were applied in the paper [44], employing nonlinear fuzzy-parameter PDE to option pricing and hedging. Ref. [45] introduced the fractional Brownian motion to the problem of option valuation in a fuzzy environment. Finally, an approach based on fuzzy geometric Brownian motion, fuzzy lognormal distribution and fuzzy Ito integral was proposed in [46].

Among other solutions to the option pricing problem, applying discrete time stochastic processes or type-2 fuzzy logic, one can mention the papers [47,48,49,50].

## 3. The Proposed Model Underlying Dynamics of Electricity Spot Prices

This section contains the proposed model of electricity spot prices. The mixed-exponential distribution of jumps was used in [1] to model equities’ prices. However, its application to the mean-reverting jump-diffusion process of the dynamics of electricity prices is novel. A distinctive feature of the mixed-exponential distribution is the ability to approximate the distributions from a large class, including the long-tailed Pareto and Weibull, arbitrarily closely (see [51]).

We assume that a time horizon $T=\left[0,T^{*}\right]$ is finite, i.e., $T^{*}>0$. Let $\left(\Omega ,F,{\left(F_{t}\right)}_{t\in T},P\right)$ be a probability space with filtration satisfying the usual assumptions, on which all the considered processes and random variables are defined. The stochastic process $S_{t}$ of spot price has the following form:

(1) $$S_{t}=\exp\left(g\left(t\right)+X_{t}\right),$$

(2) $$dX_{t}=-\mu X_{t}dt+\sigma dW_{t}+dJ_{t},$$

deterministic function g(t) describes a seasonality estimated from historical data, μ,σ>0, ${\left(W_{t}\right)}_{t\in T}$ denotes a (F,P) Wiener process, and ${\left(J_{t}\right)}_{t\in T}$ a (F,P) compound Poisson process given by $J_{t}=\sum_{i=1}^{N_{t}}Z_{i},\;t\in T,$ where $N_{t}$ is a Poisson process with an intensity λ>0, ${\left\{Z_{i}\right\}}_{i\in N}$ are i.i.d. jump magnitudes of the mixed-exponential distribution, i.e., with density of the form

(3) $$f\left(z\right)=q_{d}\sum_{i=1}^{m}q_{i}\xi_{i}e^{\xi_{i}z}{𝟙}_{\left\{z<0\right\}}+p_{u}\sum_{j=1}^{n}p_{j}\eta_{j}e^{-\eta_{j}z}{𝟙}_{\left\{z>0\right\}},$$

$q_{d},p_{u}\geq 0,\;\;\;q_{d}+p_{u}=1,\;\;q_{i},p_{j}\in \left(-\infty ,\infty \right),\;\sum_{i=1}^{m}q_{i}=\sum_{j=1}^{n}p_{j}=1,\;\xi_{i}>0,$ $\eta_{j}>1$. In the formula above, $q_{d}$ and $p_{u}$ are the probabilities of negative and positive jumps, respectively.

In the following part of the paper we will use the notion [N]={1,2,…,N} for each positive integer *N*. A necessary condition for f(z) to be a the probability density function is

$$q_{1},p_{1}>0,\;\;\sum_{i=1}^{m}q_{i}\xi_{i}\geq 0,\;\;\sum_{j=1}^{n}p_{j}\eta_{j}\geq 0,$$

whereas $\sum_{i=1}^{k}q_{i}\xi_{i}\geq 0,\;\;\sum_{j=1}^{l}p_{j}\eta_{j}\geq 0$, k∈[m], l∈[n] is one of sufficient conditions. If $q_{i}\geq 0$, $p_{j}\geq 0$ for each i∈[m], j∈[n], then the distribution mentioned above is hyperexponential. For further detail, we refer the reader to [1].

Ito’s lemma implies that the following stochastic differential equation

(4) $$dS_{t}=\mu \left(\rho \left(t\right)-\ln S_{t}\right)S_{t}dt+\sigma S_{t}dW_{t}+S_{t}\left(e^{Z_{N_{t}}}-1\right)dN_{t},$$

where $\rho \left(t\right)=\frac{1}{\mu }\left(\frac{dg(t)}{dt}+\frac{1}{2}\sigma^{2}\right)+g\left(t\right),\;\;Z_{0}=0$ describes $S_{t}$.

Finally, we assume that $F=F_{T^{*}}$, filtration ${\left(F_{t}\right)}_{t\in T}$ is generated by processes *W* and *J*, and is augmented to encompass P-null sets from F.

## 4. Pricing Forward Contracts with Crisp Parameters

We denote by ${\left(I_{t}\right)}_{t\in T}$ an independent increments process and by (γ,C,l(du,dz)) under P its semimartingale characteristics. In this paper, we assume that the risk-neutral measure Q, equivalent to P, is obtained by the Esscher transform, i.e.,

(5) $$\frac{dQ}{dP}{|}_{F_{t}}={\hat{Z}}^{\theta }\left(t\right){\bar{Z}}^{\theta }\left(t\right),$$

for a 2-dimensional vector $\theta \left(t\right)=(\hat{\theta }\left(t\right),\bar{\theta }\left(t\right))$ of R-valued continuous functions on T,

$${\hat{Z}}^{\theta }\left(t\right)=\exp\left(\int_{0}^{t}\hat{\theta }\left(s\right)dW_{s}-\frac{1}{2}\int_{0}^{t}{\hat{\theta }}^{2}\left(s\right)ds\right),$$

$$\begin{array}{cc} & {\bar{Z}}^{\theta }\left(t\right)=\exp\left(\int_{0}^{t}\bar{\theta }\left(s\right)dI_{s}-\phi \left(0,t,\bar{\theta }\left(\cdot \right)\right)\right), \end{array}$$

$$\begin{array}{cc} & \underset{t\in T}{\sup}\left|\bar{\theta }\left(t\right)\right|\leq c\;\;\mathrm{and}\;\;\int_{T}\int_{1}^{\infty }\left\{e^{cz}-1\right\}l\left(dz,du\right)<\infty , \end{array}$$

$c\in R_{+}$, and

$$\begin{array}{cc} & \phi \left(0,t,\bar{\theta }\left(\cdot \right)\right)=\int_{0}^{t}\bar{\theta }\left(u\right)d\gamma \left(u\right)+\frac{1}{2}\int_{0}^{t}{\bar{\theta }}^{2}\left(u\right)dC\left(u\right)+\\ & \int_{0}^{t}\int_{R}\left\{e^{\bar{\theta }\left(u\right)z}-1-\bar{\theta }\left(u\right)z{𝟙}_{|z|<1}\right\}l\left(dz,du\right). \end{array}$$

The following proposition holds.

**Proposition** **1.** *Process $W_{t}^{Q}$ given by the equality*

$$W_{t}^{Q}=W_{t}-\int_{0}^{t}\hat{\theta }\left(s\right)ds,\;\;t\in T,$$

*is a Q-Brownian motion and the independent increments process I under Q has drift*

$$\gamma \left(t\right)+\int_{0}^{t}\int_{|z|<1}z\left\{e^{\bar{\theta }\left(u\right)z}-1\right\}l\left(dz,du\right)+\int_{0}^{t}{\bar{\theta }}^{2}\left(u\right)dC\left(u\right)$$

*and predictable compensator measure $e^{\bar{\theta }\left(t\right)z}l\left(dz,dt\right)$.*

It was proved in [52] in a more general case.

**Definition** **1** **(Forward price).** *A forward price $F^{Q}\left(t,T\right)$ at time t is given by a conditional expected value of a spot price in the future time T:*

(6) $$F^{Q}\left(t,T\right)=E^{Q}\left[S_{T}|F_{t}\right],$$

*where Q is the risk-neutral measure.*

The risk-neutral measure is described by the Esscher transform (5) for *W* and I=J, where we assume that $\hat{\theta }\equiv {\hat{\theta }}_{0}\in R$, $\bar{\theta }\equiv {\bar{\theta }}_{0}\in R$. ${\hat{\theta }}_{0}$ and ${\bar{\theta }}_{0}$ are called the market price of diffusion risk and the market price of jump risk, respectively. We additionally assume that $\max\left(-\min_{i\in [m]}\xi_{i},-\min_{j\in [n]}\eta_{j}\right)<{\bar{\theta }}_{0}<\min_{j\in [n]}\eta_{j}-1.$

**Theorem** **1.** *In a crisp case, the forward price has the following analytical form:*

(7) $$\begin{array}{cc} F^{Q}\left(t,T\right) & =E^{Q}\left[S_{T}|F_{t}\right]=e^{g(T)}{\left(\frac{S_{t}}{e^{g(t)}}\right)}^{m_{t,T}}\cdot \\ & \exp\left[\frac{\sigma \left(1-m_{t,T}\right)\left(\sigma \left(1+m_{t,T}\right)+4{\hat{\theta }}_{0}\right)}{4\mu }\right]\cdot \\ & \exp\left[\frac{\lambda^{Q}}{\mu }\left(q_{d}^{Q}\sum_{i=1}^{m}q_{i}^{Q}\ln\frac{\xi_{i}^{Q}+m_{t,T}}{\xi_{i}^{Q}+1}+p_{u}^{Q}\sum_{j=1}^{n}p_{j}^{Q}\ln\frac{\eta_{j}^{Q}-m_{t,T}}{\eta_{j}^{Q}-1}\right)\right],\;t\in T, \end{array}$$

*where*

$$\begin{array}{cc} \; & \xi_{i}^{Q}=\xi_{i}+{\bar{\theta }}_{0},\;q_{i}^{Q}=\frac{q_{i}\xi_{i}}{\xi_{i}^{Q}\sum_{i=1}^{m}\frac{q_{i}\xi_{i}}{\xi_{i}^{Q}}},\;i\in \left[m\right],\;\eta_{j}^{Q}=\eta_{j}-{\bar{\theta }}_{0},\;p_{j}^{Q}=\frac{p_{j}\eta_{j}}{\eta_{j}^{Q}\sum_{j=1}^{n}\frac{p_{j}\eta_{j}}{\eta_{j}^{Q}}},\;j\in \left[n\right],\\ \; & q_{d}^{Q}=q_{d}\frac{\lambda }{\lambda^{Q}}\sum_{i=1}^{m}\frac{q_{i}\xi_{i}}{\xi_{i}^{Q}},\;p_{u}^{Q}=p_{u}\frac{\lambda }{\lambda^{Q}}\sum_{j=1}^{n}\frac{p_{j}\eta_{j}}{\eta_{j}^{Q}},\;m_{t,T}=e^{-\mu (T-t)},\\ & \lambda^{Q}=\lambda \left[q_{d}\sum_{i=1}^{m}\frac{q_{i}\xi_{i}}{\xi_{i}^{Q}}+p_{u}\sum_{j=1}^{n}\frac{p_{j}\eta_{j}}{\eta_{j}^{Q}}\right]. \end{array}$$

**Proof.**  Let us consider the process $Y_{t}=\ln\left(S_{t}\right)$ and revise the Equation (4). We apply Ito’s lemma for $Y_{t}$ and the Esscher transform to change the physical probability measure P to the equivalent risk-neutral measure Q. By Proposition 1

(8) $$dY_{t}=\mu \left(\rho^{Q}\left(t\right)-Y_{t}\right)dt+\sigma dW_{t}^{Q}+Z_{N_{t}^{Q}}^{Q}dN_{t}^{Q}$$

for $\rho^{Q}\left(t\right)=\frac{1}{\mu }\frac{dg(t)}{dt}+g\left(t\right)+\frac{\sigma {\hat{\theta }}_{0}}{\mu }$ and a Q-Poisson process $N^{Q}$ with intensity

$$\lambda^{Q}=\lambda \int_{R}e^{{\bar{\theta }}_{0}z}f\left(z\right)dz=\lambda \left[q_{d}\sum_{i=1}^{m}\frac{q_{i}\xi_{i}}{\xi_{i}^{Q}}+p_{u}\sum_{j=1}^{n}\frac{p_{j}\eta_{j}}{\eta_{j}^{Q}}\right],$$

independent from a Q-Brownian motion $W^{Q}$. Moreover, in the formula above, ${\left\{Z_{i}^{Q}\right\}}_{i\in N}$ are independent identically distributed random variables with the mixed-exponential distribution and probability density function

(9) $$f^{Q}\left(z\right)=q_{d}^{Q}\sum_{i=1}^{m}q_{i}^{Q}\xi_{i}^{Q}e^{\xi_{i}^{Q}z}{𝟙}_{\left\{z<0\right\}}+p_{u}^{Q}\sum_{j=1}^{n}p_{j}^{Q}\eta_{j}^{Q}e^{-\eta_{j}^{Q}z}{𝟙}_{\left\{z>0\right\}}$$

and

$$\begin{array}{cc} \; & \xi_{i}^{Q}=\xi_{i}+{\bar{\theta }}_{0},\;q_{i}^{Q}=\frac{q_{i}\xi_{i}}{\xi_{i}^{Q}\sum_{i=1}^{m}\frac{q_{i}\xi_{i}}{\xi_{i}^{Q}}},\;i\in \left[m\right],\;\eta_{j}^{Q}=\eta_{j}-{\bar{\theta }}_{0},\;p_{j}^{Q}=\frac{p_{j}\eta_{j}}{\eta_{j}^{Q}\sum_{j=1}^{n}\frac{p_{j}\eta_{j}}{\eta_{j}^{Q}}},\;j\in \left[n\right],\\ \; & q_{d}^{Q}=q_{d}\frac{\lambda }{\lambda^{Q}}\sum_{i=1}^{m}\frac{q_{i}\xi_{i}}{\xi_{i}^{Q}},\;p_{u}^{Q}=p_{u}\frac{\lambda }{\lambda^{Q}}\sum_{j=1}^{n}\frac{p_{j}\eta_{j}}{\eta_{j}^{Q}}. \end{array}$$

We multiply both sides of (8) by $m_{t,T}$ and integrate from *t* to T. Consequently, the equation converts to

(10) $$\begin{array}{c} \int_{t}^{T}m_{s,T}dY_{s}=\int_{t}^{T}m_{s,T}dg\left(s\right)+\int_{t}^{T}\mu m_{s,T}g\left(s\right)ds-\int_{t}^{T}\mu m_{s,T}Y_{s}ds+\\ \int_{t}^{T}\sigma {\hat{\theta }}_{0}m_{s,T}ds+\int_{t}^{T}\sigma m_{s,T}dW_{s}^{Q}+\int_{t}^{T}m_{s,T}Z_{N_{s}^{Q}}^{Q}dN_{s}^{Q}. \end{array}$$

Because

(11) $$\begin{array}{cc} & -\int_{t}^{T}\mu m_{s,T}Y_{s}ds=m_{t,T}Y_{t}-Y_{T}+\int_{t}^{T}m_{s,T}dY_{s} \end{array}$$

and

(12) $$\begin{array}{cc} & \int_{t}^{T}\mu m_{s,T}g\left(s\right)ds=g\left(T\right)-m_{t,T}g\left(t\right)-\int_{t}^{T}m_{s,T}dg\left(s\right), \end{array}$$

we may write

(13) $$\begin{array}{c} Y_{T}=g\left(T\right)+\left(Y_{t}-g\left(t\right)\right)m_{t,T}+\int_{t}^{T}\sigma {\hat{\theta }}_{0}\;m_{s,T}ds+\int_{t}^{T}\sigma m_{s,T}dW_{s}^{Q}+\\ \int_{t}^{T}m_{s,T}Z_{N_{s}^{Q}}^{Q}dN_{s}^{Q}. \end{array}$$

By the Dynkin lemma and an approach similar as in [53], we obtain

(14) $$\begin{array}{cc} \; & E^{Q}\left[\exp\left(\int_{t}^{T}\sigma m_{s,T}dW_{s}^{Q}+\int_{t}^{T}m_{s,T}Z_{N_{s}^{Q}}^{Q}dN_{s}^{Q}\right)|F_{t}\right]=\\ \; & E^{Q}\left[\exp\left(\int_{t}^{T}\sigma m_{s,T}dW_{s}^{Q}\right)|F_{t}\right]E^{Q}\left[\exp\left(\int_{t}^{T}m_{s,T}Z_{N_{s}^{Q}}^{Q}dN_{s}^{Q}\right)|F_{t}\right]. \end{array}$$

By (13) and (14), taking into account $S_{T}=e^{Y_{T}}$ and introducing notation $G\left(t\right)=e^{g(t)},$

(15) $$\begin{array}{cc} & F^{Q}\left(t,T\right)=E^{Q}\left[S_{T}|F_{t}\right]=G\left(T\right){\left(\frac{S_{t}}{G(t)}\right)}^{m_{t,T}}\exp\left(\int_{t}^{T}\sigma {\hat{\theta }}_{0}\;m_{s,T}ds\right)\cdot \\ & E^{Q}\left[\exp\left(\int_{t}^{T}\sigma m_{s,T}dW_{s}^{Q}\right)|F_{t}\right]E^{Q}\left[\exp\left(\int_{t}^{T}m_{s,T}Z_{N_{s}^{Q}}^{Q}dN_{s}^{Q}\right)|F_{t}\right]=\\ & G\left(T\right){\left(\frac{S_{t}}{G(t)}\right)}^{m_{t,T}}\exp\left(\int_{t}^{T}\sigma m_{s,T}\left(\frac{1}{2}\sigma m_{s,T}+{\hat{\theta }}_{0}\right)ds\right)\cdot \\ & E^{Q}\left[\exp\left(\int_{t}^{T}m_{s,T}Z_{N_{s}^{Q}}^{Q}dN_{s}^{Q}\right)|F_{t}\right]=\\ \; & G\left(T\right){\left(\frac{S_{t}}{G(t)}\right)}^{m_{t,T}}\exp\left(\frac{\sigma \left(1-m_{t,T}\right)}{4\mu }\left(\sigma \left(1+m_{t,T}\right)+4{\hat{\theta }}_{0}\right)\right)\cdot \\ & E^{Q}\left[\exp\left(\int_{t}^{T}m_{s,T}Z_{N_{s}^{Q}}^{Q}dN_{s}^{Q}\right)|F_{t}\right], \end{array}$$

inasmuch as

(16) $$\begin{array}{cc} & E^{Q}\left[\exp\left(\int_{t}^{T}\sigma m_{s,T}dW_{s}^{Q}\right)|F_{t}\right]=\exp\left(\frac{1}{2}\int_{t}^{T}\sigma^{2}e^{-2\mu (T-s)}ds\right). \end{array}$$

Similarly as in [20] (part A of Appendix), we have the equality

(17) $$\begin{array}{cc} & E^{Q}\left[\exp\left(\int_{t}^{T}m_{s,T}Z_{N_{s}^{Q}}^{Q}dN_{s}^{Q}\right)|F_{t}\right]=\exp\left(\int_{t}^{T}\left(E^{Q}\left[e^{m_{s,T}Z_{N_{s}^{Q}}^{Q}}\right]-1\right)\lambda^{Q}ds\right). \end{array}$$

Straightforward computations give

(18) $$\begin{array}{cc} & E^{Q}\left[e^{m_{s,T}Z_{N_{s}^{Q}}^{Q}}\right]=q_{d}^{Q}\sum_{i=1}^{m}\frac{q_{i}^{Q}\xi_{i}^{Q}e^{\mu (T-s)}}{\xi_{i}^{Q}e^{\mu (T-s)}+1}+p_{u}^{Q}\sum_{j=1}^{n}\frac{p_{j}^{Q}\eta_{j}^{Q}e^{\mu (T-s)}}{\eta_{j}^{Q}e^{\mu (T-s)}-1}. \end{array}$$

Therefore,

(19) $$\begin{array}{cc} E^{Q} & \left[\exp\left(\int_{t}^{T}m_{s,T}Z_{N_{s}^{Q}}^{Q}dN_{s}^{Q}\right)|F_{t}\right]=\\ \exp & \left[q_{d}^{Q}\sum_{i=1}^{m}q_{i}^{Q}\int_{t}^{T}\frac{\xi_{i}^{Q}e^{\mu (T-s)}}{\xi_{i}^{Q}e^{\mu (T-s)}+1}\lambda^{Q}ds+p_{u}^{Q}\sum_{j=1}^{n}p_{j}^{Q}\int_{t}^{T}\frac{\eta_{j}^{Q}e^{\mu (T-s)}}{\eta_{j}^{Q}e^{\mu (T-s)}-1}\lambda^{Q}ds-\right.\\ \; & \lambda^{Q}\left(T-t\right)]=\\ \exp & \left[\frac{\lambda^{Q}}{\mu }\left(q_{d}^{Q}\sum_{i=1}^{m}q_{i}^{Q}\ln\frac{\xi_{i}^{Q}+m_{t,T}}{\xi_{i}^{Q}+1}+p_{u}^{Q}\sum_{j=1}^{n}p_{j}^{Q}\ln\frac{\eta_{j}^{Q}-m_{t,T}}{\eta_{j}^{Q}-1}\right)\right], \end{array}$$

which completes the proof. □

**Definition** **2** **(Forward contract).** *Let us denote by the symbols $T_{1}<\ldots <T_{N}\in T$ and K electricity delivery days and a delivery price, respectively. A forward contract is a derivative financial instrument which enables to receive electricity on days $T_{1},T_{2},\ldots ,T_{N}$ for K, i.e., which pays the difference*

(20) $$\frac{1}{N}\sum_{i=1}^{N}S_{T_{i}}-K.$$

We denote by the symbol $F_{t}^{Q}$ the value of the forward contract mentioned above. Theorem 1 enables obtaining the analytical form of $F_{t}^{Q}$, using the following formula:

(21) $$F_{t}^{Q}=E^{Q}\left[\frac{1}{N}\sum_{i=1}^{N}S_{T_{i}}-K|F_{t}\right]=\frac{1}{N}\sum_{i=1}^{N}F^{Q}\left(t,T_{i}\right)-K$$

and adapting the formula (7) for each element of the sum.

**Definition** **3** **(Forward price of a forward contract).** *A forward price $K_{t}$ of a forward contract at time t is a value of a delivery price K introduced in Definition 2, for which $F_{t}^{Q}=\frac{1}{N}\sum_{i=1}^{N}F^{Q}\left(t,T_{i}\right)-K_{t}=0.$*

## 5. The Adjusted Fuzzy Decision-Making Method

This section is devoted to the fuzzy approach to pricing forward contracts. For a brief summary of fuzzy numbers theory and interval arithmetic we refer the readers to [13,31]. We restrict our attention only to necessary notations.

We denote by R, $B\left(R\right)$ and $F\left(R\right)$ the set of real numbers, the σ-field of Borel subsets of R and the set of fuzzy numbers, respectively.

For $\tilde{a}\in F\left(R\right)$, $\mu_{\tilde{a}}:R\mapsto \left[0,1\right]$ denotes its membership function. For arbitrary α∈[0,1], ${\tilde{a}}_{\alpha }=\left[{\tilde{a}}_{\alpha }^{L},{\tilde{a}}_{\alpha }^{U}\right]$, where $-\infty <{\tilde{a}}_{\alpha }^{L}\leq {\tilde{a}}_{\alpha }^{U}<\infty$, are its α-level sets.

Assume that L,R:[0,1]↦[0,1] are continuous and strictly decreasing functions such that L(0)=R(0)=1, L(1)=R(1)=0 and $a_{1},a_{2},a_{3}\in R$ satisfy the inequality: $a_{1}<a_{2}<a_{3}$. An element $\tilde{a}$ of $F\left(R\right)$ is called an L-R (left-right) fuzzy number, if

$$\mu_{\tilde{a}}\left(x\right)=\left\{\begin{array}{cc} L\left(\frac{a_{2}-x}{a_{2}-a_{1}}\right) & \mathrm{for}\;a_{1}\leq x\leq a_{2};\\ R\left(\frac{x-a_{2}}{a_{3}-a_{2}}\right) & \mathrm{for}\;a_{2}\leq x\leq a_{3};\\ 0 & \mathrm{otherwise}. \end{array}\right.$$

If L(y)=R(y)=1−y, $\tilde{a}$ is called a triangular fuzzy number and is denoted by $\tilde{a}=\left(a_{1},a_{2},a_{3}\right)$.

A function $\tilde{X}:\Omega \mapsto F\left(R\right)$, where $\left(\Omega ,F\right)$ is a measurable space, is called a fuzzy random variable (see, e.g., [54]) if for each α∈[0,1]

$$\left\{\left(\omega ,x\right):\tilde{X}\left(\omega \right)\left(x\right)\geq \alpha \right\}\in F\times B\left(R\right).$$

We also use the notation ⊕, ⊖, ⊗ and ⊘ for the arithmetic operations between fuzzy numbers, defined by the extension principle (see, e.g., [55,56,57]) and corresponding arithmetic operations +,−,×, between real numbers, respectively.

Furthermore, the symbols ${⊕}_{\mathrm{int}}$, ${⊖}_{\mathrm{int}}$, ${⊗}_{\mathrm{int}}$ and ${⊘}_{\mathrm{int}}$ denote the arithmetic operations between closed intervals.

It was discussed in [13,31] that there exists a correspondence between α-level sets of results of fuzzy arithmetic operations on fuzzy arguments and interval arithmetic operations on α-level sets of these arguments.

Weighted crisp possibilistic mean value of a fuzzy number was introduced in [58]. Let $\tilde{a}\in F\left(R\right)$. An increasing, nonnegative function f:[0,1]↦R is called a weighting function if it satisfies the following normalization condition

(22) $$\int_{0}^{1}f\left(\alpha \right)d\alpha =1.$$

The lower and upper weighted possibilistic mean values of $\tilde{a}$ are given by

$$\begin{array}{c} M_{*}\left(\tilde{a}\right)=\int_{0}^{1}{\tilde{a}}_{\alpha }^{L}f\left(\alpha \right)d\alpha ,\;M^{*}\left(\tilde{a}\right)=\int_{0}^{1}{\tilde{a}}_{\alpha }^{U}f\left(\alpha \right)d\alpha , \end{array}$$

respectively, whereas the weighted possibilistic mean of $\tilde{a}$ is the arithmetic mean

$$\bar{M}\left(\tilde{a}\right)=\frac{M_{*}\left(\tilde{a}\right)+M^{*}\left(\tilde{a}\right)}{2}.$$

In the further part of the paper we assume that f(α)=2α.

The uncertainty of the model parameters in this paper is described by L-R fuzzy numbers. They can be obtained, i.a., from experts (see, e.g., [13,59,60]). The symbol ^~^ above fuzzy parameters is used to indicate their fuzziness.

A similar technique was applied for the first time to option pricing in the Black–Scholes model in [31].

The parameters μ, σ, λ, $\xi ={\left\{\xi_{i}\right\}}_{i\in [m]}$, $\eta ={\left\{\eta_{j}\right\}}_{j\in [n]}$ of the crisp model are replaced by their counterparts $\tilde{\mu },\tilde{\sigma },\tilde{\lambda }$, $\tilde{\xi }={\left\{{\tilde{\xi }}_{i}\right\}}_{i\in [m]}$, $\tilde{\eta }={\left\{{\tilde{\eta }}_{j}\right\}}_{j\in [n]}$ in the form of L-R fuzzy numbers. We also treat values of process ${\tilde{S}}_{t}$, t∈T, as fuzzy random variables. We additionally assume the positivity of $\tilde{\mu },\tilde{\sigma },\tilde{\lambda }$, ${\tilde{\xi }}_{i}⊕{\bar{\theta }}_{0},i\in \left[m\right]$, ${\tilde{\eta }}_{j}⊖{\bar{\theta }}_{0}⊖1,j\in \left[n\right]$ and ${\tilde{S}}_{t}$, t∈T, i.e., the positivity of their membership functions for positive arguments.

Finally, we make the assumption that the fuzzy numbers $\overset{m}{\underset{i=1}{⨁}}q_{i}⊗{\tilde{\xi }}_{i}⊘\left({\tilde{\xi }}_{i}⊕{\bar{\theta }}_{0}\right)$, $\overset{n}{\underset{j=1}{⨁}}p_{j}⊗{\tilde{\eta }}_{j}⊘\left({\tilde{\eta }}_{j}⊖{\bar{\theta }}_{0}\right)$ are positive. This assumption is always satisfied, when the distribution of jumps in the crisp model is hyperexponential.

To shorten notation we introduce the set of symbols Σ={L,U} and the operator ${}^{\prime }:\Sigma \mapsto \Sigma$ given by: $L^{\prime }=U$, $U^{\prime }=L$.

**Theorem** **2.** *The fuzzy forward price is given by the following analytical formula:*

(23) $$\begin{array}{cc} & {\tilde{F}}^{Q}\left(t,T\right)=\exp\left[{\tilde{m}}_{t,T}⊗\ln{\tilde{S}}_{t}⊕g\left(T\right)⊖g\left(t\right)⊗{\tilde{m}}_{t,T}⊕{\tilde{\Gamma }}_{t,T}⊘\tilde{M}\right],\;t\in T, \end{array}$$

*where*

(24) $$\begin{array}{c} \begin{array}{cc} \; & {\tilde{m}}_{t,T}=e^{-\left(T-t\right)⊗\tilde{\mu }},\;\tilde{M}=4⊗\tilde{\mu },\;{\tilde{\xi }}_{i}^{Q}={\tilde{\xi }}_{i}⊕{\bar{\theta }}_{0},\;i\in \left[m\right],\;{\tilde{\eta }}_{j}^{Q}={\tilde{\eta }}_{j}⊖{\bar{\theta }}_{0}\;j\in \left[n\right],\\ \; & {\tilde{\lambda }}^{Q}=\tilde{\lambda }⊗\left[q_{d}⊗\overset{m}{\underset{i=1}{⨁}}q_{i}⊗{\tilde{\xi }}_{i}⊘{\tilde{\xi }}_{i}^{Q}⊕p_{u}⊗\overset{n}{\underset{j=1}{⨁}}p_{j}⊗{\tilde{\eta }}_{j}⊘{\tilde{\eta }}_{j}^{Q}\right],\\ \; & {\tilde{q}}_{d}^{Q}=\left(q_{d}⊗\tilde{\lambda }⊘{\tilde{\lambda }}^{Q}\right)⊗\overset{m}{\underset{i=1}{⨁}}q_{i}⊗{\tilde{\xi }}_{i}⊘{\tilde{\xi }}_{i}^{Q},\\ \; & {\tilde{p}}_{u}^{Q}=\left(p_{u}⊗\tilde{\lambda }⊘{\tilde{\lambda }}^{Q}\right)⊗\overset{n}{\underset{j=1}{⨁}}p_{j}⊗{\tilde{\eta }}_{j}⊘{\tilde{\eta }}_{j}^{Q},\\ \; & {\tilde{q}}_{i}^{Q}=\left(q_{i}⊗{\tilde{\xi }}_{i}⊘{\tilde{\xi }}_{i}^{Q}\right)⊘\left(\overset{m}{\underset{i=1}{⨁}}q_{i}⊗{\tilde{\xi }}_{i}⊘{\tilde{\xi }}_{i}^{Q}\right),\;i\in \left[m\right],\\ \; & {\tilde{p}}_{j}^{Q}=\left(p_{j}⊗{\tilde{\eta }}_{j}⊘{\tilde{\eta }}_{j}^{Q}\right)⊘\left(\overset{n}{\underset{j=1}{⨁}}p_{j}⊗{\tilde{\eta }}_{j}⊘{\tilde{\eta }}_{j}^{Q}\right),\;j\in \left[n\right],\\ \; & {\tilde{\Gamma }}_{t,T}={\tilde{\Gamma }}_{1,t,T}⊕{\tilde{\Gamma }}_{2,t,T},\;{\tilde{\Gamma }}_{1,t,T}={\tilde{\gamma }}_{1,1,t,T}⊗{\tilde{\gamma }}_{1,2,t,T},\\ \; & {\tilde{\gamma }}_{1,1,t,T}=\tilde{\sigma }⊗\left(1⊖{\tilde{m}}_{t,T}\right),\;{\tilde{\gamma }}_{1,2,t,T}=\tilde{\sigma }⊗\left(1⊕{\tilde{m}}_{t,T}\right)⊕4⊗{\hat{\theta }}_{0},\\ \; & {\tilde{\Gamma }}_{2,t,T}=4⊗{\tilde{\lambda }}^{Q}⊗{\tilde{\gamma }}_{2,t,T},\;{\tilde{\gamma }}_{2,t,T}={\tilde{q}}_{d}^{Q}⊗{\tilde{\Lambda }}_{t,T}^{\prime }⊕{\tilde{p}}_{u}^{Q}⊗{\tilde{\Lambda }}_{t,T}^{\prime \prime },\\ \; & {\tilde{\Lambda }}_{t,T}^{\prime }=\overset{m}{\underset{i=1}{⨁}}{\tilde{\Lambda }}_{i,t,T}^{\prime },\;{\tilde{\Lambda }}_{t,T}^{\prime \prime }=\overset{n}{\underset{j=1}{⨁}}{\tilde{\Lambda }}_{j,t,T}^{\prime \prime },\\ \; & {\tilde{\Lambda }}_{i,t,T}^{\prime }={\tilde{q}}_{i}^{Q}⊗{\tilde{\Lambda }}_{i,t,T}^{1},\;{\tilde{\Lambda }}_{i,t,T}^{1}=\ln\left(\left({\tilde{\xi }}_{i}^{Q}⊕{\tilde{m}}_{t,T}\right)⊘\left({\tilde{\xi }}_{i}^{Q}⊕1\right)\right),\;i\in \left[m\right],\\ \; & {\tilde{\Lambda }}_{j,t,T}^{\prime \prime }={\tilde{p}}_{j}^{Q}⊗{\tilde{\Lambda }}_{j,t,T}^{2},\;{\tilde{\Lambda }}_{j,t,T}^{2}=\ln\left(\left({\tilde{\eta }}_{j}^{Q}⊖{\tilde{m}}_{t,T}\right)⊘\left({\tilde{\eta }}_{j}^{Q}⊖1\right)\right),\;j\in \left[n\right]. \end{array} \end{array}$$

*Furthermore, for each α∈[0,1] and Ξ∈Σ*

(25) $$\begin{array}{c} \begin{array}{cc} & {\left({\tilde{F}}^{Q}\left(t,T\right)\right)}_{\alpha }^{\Xi }=\exp\left[{\left({\tilde{m}}_{t,T}\right)}_{\alpha }^{\Xi }\ln{\left({\tilde{S}}_{t}\right)}_{\alpha }^{\Xi }+g\left(T\right)-{\left(g\left(t\right){⊗}_{int}{\left({\tilde{m}}_{t,T}\right)}_{\alpha }\right)}^{\Xi^{\prime }}+\right.\\ \; & \left.{\left({\left({\tilde{\Gamma }}_{t,T}\right)}_{\alpha }{⊘}_{int}{\tilde{M}}_{\alpha }\right)}^{\Xi }\right],\;t\in T, \end{array} \end{array}$$

*where*

(26) $$\begin{array}{c} \begin{array}{cc} \; & {\left({\tilde{m}}_{t,T}\right)}_{\alpha }^{\Xi }=e^{-\left(T-t\right){\tilde{\mu }}_{\alpha }^{\Xi^{\prime }}},\;{\tilde{M}}_{\alpha }^{\Xi }=4{\tilde{\mu }}_{\alpha }^{\Xi },\\ \; & {\left({\tilde{\xi }}_{i}^{Q}\right)}_{\alpha }^{\Xi }={\left({\tilde{\xi }}_{i}\right)}_{\alpha }^{\Xi }+{\bar{\theta }}_{0},\;{\left({\tilde{q}}_{i}^{Q}\right)}_{\alpha }^{\Xi }=\frac{q_{i}{\left({\tilde{\xi }}_{i}\right)}_{\alpha }^{\Xi }/{\left({\tilde{\xi }}_{i}^{Q}\right)}_{\alpha }^{\Xi^{\prime }}}{\sum_{i=1}^{m}q_{i}{\left({\tilde{\xi }}_{i}\right)}_{\alpha }^{\Xi^{\prime }}/{\left({\tilde{\xi }}_{i}^{Q}\right)}_{\alpha }^{\Xi }},\;i\in \left[m\right],\\ \; & {\left({\tilde{\eta }}_{j}^{Q}\right)}_{\alpha }^{\Xi }={\left({\tilde{\eta }}_{j}\right)}_{\alpha }^{\Xi }-{\bar{\theta }}_{0},\;{\left({\tilde{p}}_{j}^{Q}\right)}_{\alpha }^{\Xi }=\frac{p_{j}{\left({\tilde{\eta }}_{j}\right)}_{\alpha }^{\Xi }/{\left({\tilde{\eta }}_{j}^{Q}\right)}_{\alpha }^{\Xi^{\prime }}}{\sum_{j=1}^{n}p_{j}{\left({\tilde{\eta }}_{j}\right)}_{\alpha }^{\Xi^{\prime }}/{\left({\tilde{\eta }}_{j}^{Q}\right)}_{\alpha }^{\Xi }},\;j\in \left[n\right],\\ \; & {\left({\tilde{\lambda }}^{Q}\right)}_{\alpha }^{\Xi }={\tilde{\lambda }}_{\alpha }^{\Xi }\left(q_{d}\sum_{i=1}^{m}q_{i}{\left({\tilde{\xi }}_{i}\right)}_{\alpha }^{\Xi }/{\left({\tilde{\xi }}_{i}^{Q}\right)}_{\alpha }^{\Xi^{\prime }}+p_{u}\sum_{j=1}^{n}p_{j}{\left({\tilde{\eta }}_{j}\right)}_{\alpha }^{\Xi }/{\left({\tilde{\eta }}_{j}^{Q}\right)}_{\alpha }^{\Xi^{\prime }}\right),\\ \; & {\left({\tilde{q}}_{d}^{Q}\right)}_{\alpha }^{\Xi }=q_{d}{\left(\tilde{\lambda }\right)}_{\alpha }^{\Xi }/{\left({\tilde{\lambda }}^{Q}\right)}_{\alpha }^{\Xi^{\prime }}\sum_{i=1}^{m}q_{i}{\left({\tilde{\xi }}_{i}\right)}_{\alpha }^{\Xi }/{\left({\tilde{\xi }}_{i}^{Q}\right)}_{\alpha }^{\Xi^{\prime }},\\ \; & {\left({\tilde{p}}_{u}^{Q}\right)}_{\alpha }^{\Xi }=p_{u}{\left(\tilde{\lambda }\right)}_{\alpha }^{\Xi }/{\left({\tilde{\lambda }}^{Q}\right)}_{\alpha }^{\Xi^{\prime }}\sum_{j=1}^{n}p_{j}{\left({\tilde{\eta }}_{j}\right)}_{\alpha }^{\Xi }/{\left({\tilde{\eta }}_{j}^{Q}\right)}_{\alpha }^{\Xi^{\prime }}. \end{array} \end{array}$$

(27) $$\begin{array}{c} \begin{array}{cc} \; & {\left({\tilde{\Gamma }}_{t,T}\right)}_{\alpha }^{\Xi }={\left({\tilde{\Gamma }}_{1,t,T}\right)}_{\alpha }^{\Xi }+{\left({\tilde{\Gamma }}_{2,t,T}\right)}_{\alpha }^{\Xi },\;{\left({\tilde{\Gamma }}_{1,t,T}\right)}_{\alpha }^{\Xi }={\left({\left({\tilde{\gamma }}_{1,1,t,T}\right)}_{\alpha }{⊗}_{int}{\left({\tilde{\gamma }}_{1,2,t,T}\right)}_{\alpha }\right)}^{\Xi },\\ \; & {\left({\tilde{\gamma }}_{1,1,t,T}\right)}_{\alpha }^{\Xi }={\tilde{\sigma }}_{\alpha }^{\Xi }\left(1-{\left({\tilde{m}}_{t,T}\right)}_{\alpha }^{\Xi^{\prime }}\right),\;{\left({\tilde{\gamma }}_{1,2,t,T}\right)}_{\alpha }^{\Xi }={\left(\tilde{\sigma }\right)}_{\alpha }^{\Xi }\left(1+{\left({\tilde{m}}_{t,T}\right)}_{\alpha }^{\Xi }\right)+4{\hat{\theta }}_{0},\\ \; & {\left({\tilde{\Gamma }}_{2,t,T}\right)}_{\alpha }^{\Xi }=4{\left({\left({\tilde{\lambda }}^{Q}\right)}_{\alpha }{⊗}_{int}{\left({\tilde{\gamma }}_{2,t,T}\right)}_{\alpha }\right)}^{\Xi },\\ \; & {\left({\tilde{\gamma }}_{2,t,T}\right)}_{\alpha }^{\Xi }={\left({\tilde{q}}_{d}^{Q}\right)}_{\alpha }^{\Xi }{\left({\tilde{\Lambda }}_{t,T}^{\prime }\right)}_{\alpha }^{\Xi }+{\left({\tilde{p}}_{u}^{Q}\right)}_{\alpha }^{\Xi }{\left({\tilde{\Lambda }}_{t,T}^{\prime \prime }\right)}_{\alpha }^{\Xi }\\ \; & {\left({\tilde{\Lambda }}_{t,T}^{\prime }\right)}_{\alpha }^{\Xi }=\sum_{i=1}^{m}{\left({\tilde{\Lambda }}_{i,t,T}^{\prime }\right)}_{\alpha }^{\Xi },\;{\left({\tilde{\Lambda }}_{t,T}^{\prime \prime }\right)}_{\alpha }^{\Xi }=\sum_{j=1}^{n}{\left({\tilde{\Lambda }}_{j,t,T}^{\prime \prime }\right)}_{\alpha }^{\Xi }, \end{array} \end{array}$$

(28) $$\begin{array}{c} \begin{array}{cc} \; & {\left({\tilde{\Lambda }}_{i,t,T}^{\prime }\right)}_{\alpha }^{\Xi }={\left({\left({\tilde{q}}_{i}^{Q}\right)}_{\alpha }{⊗}_{int}{\left({\tilde{\Lambda }}_{i,t,T}^{1}\right)}_{\alpha }\right)}^{\Xi },\;i\in \left[m\right],\\ \; & {\left({\tilde{\Lambda }}_{i,t,T}^{1}\right)}_{\alpha }^{\Xi }=\ln\frac{{\left({\tilde{\xi }}_{i}^{Q}\right)}_{\alpha }^{\Xi }+{\left({\tilde{m}}_{t,T}\right)}_{\alpha }^{\Xi }}{{\left({\tilde{\xi }}_{i}^{Q}\right)}_{\alpha }^{\Xi^{\prime }}+1},\;i\in \left[m\right],\\ \; & {\left({\tilde{\Lambda }}_{j,t,T}^{\prime \prime }\right)}_{\alpha }^{\Xi }={\left({\left({\tilde{p}}_{j}^{Q}\right)}_{\alpha }{⊗}_{int}{\left({\tilde{\Lambda }}_{j,t,T}^{2}\right)}_{\alpha }\right)}^{\Xi },\;j\in \left[n\right],\\ \; & {\left({\tilde{\Lambda }}_{j,t,T}^{2}\right)}_{\alpha }^{\Xi }=\ln\frac{{\left({\tilde{\eta }}_{j}^{Q}\right)}_{\alpha }^{\Xi }-{\left({\tilde{m}}_{t,T}\right)}_{\alpha }^{\Xi^{\prime }}}{{\left({\tilde{\eta }}_{j}^{Q}\right)}_{\alpha }^{\Xi^{\prime }}-1},\;j\in \left[n\right]. \end{array} \end{array}$$

**Proof.**  We rewrite Formula (7) as follows:

$$\begin{array}{cc} F^{Q}\left(t,T\right)=\exp & \left[m_{t,T}\ln S_{t}+g\left(T\right)-m_{t,T}g\left(t\right)+\frac{\Gamma_{t,T}}{4\mu }\right], \end{array}$$

where

$$\begin{array}{cc} \; & \Gamma_{t,T}=\Gamma_{1,t,T}+\Gamma_{2,t,T},\;\Gamma_{1,t,T}=\sigma \left(1-m_{t,T}\right)\left(\sigma \left(1+m_{t,T}\right)+4{\hat{\theta }}_{0}\right),\\ \; & \Gamma_{2,t,T}=4\lambda^{Q}\left(q_{d}^{Q}\sum_{i=1}^{m}q_{i}^{Q}\ln\frac{\xi_{i}^{Q}+m_{t,T}}{\xi_{i}^{Q}+1}+p_{u}^{Q}\sum_{j=1}^{n}p_{j}^{Q}\ln\frac{\eta_{j}^{Q}-m_{t,T}}{\eta_{j}^{Q}-1}\right),\\ \; & \xi_{i}^{Q}=\xi_{i}+{\bar{\theta }}_{0},\;q_{i}^{Q}=\frac{\frac{q_{i}\xi_{i}}{\xi_{i}^{Q}}}{\sum_{i=1}^{m}\frac{q_{i}\xi_{i}}{\xi_{i}^{Q}}},\;i\in \left[m\right],\;\eta_{j}^{Q}=\eta_{j}-{\bar{\theta }}_{0},\;p_{j}^{Q}=\frac{\frac{p_{j}\eta_{j}}{\eta_{j}^{Q}}}{\sum_{j=1}^{n}\frac{p_{j}\eta_{j}}{\eta_{j}^{Q}}},\;j\in \left[n\right],\\ \; & m_{t,T}=e^{-\mu (T-t)},\;\lambda^{Q}=\lambda \left[q_{d}\sum_{i=1}^{m}\frac{q_{i}\xi_{i}}{\xi_{i}^{Q}}+p_{u}\sum_{j=1}^{n}\frac{p_{j}\eta_{j}}{\eta_{j}^{Q}}\right],\\ & q_{d}^{Q}=q_{d}\frac{\lambda }{\lambda^{Q}}\sum_{i=1}^{m}\frac{q_{i}\xi_{i}}{\xi_{i}^{Q}},\;p_{u}^{Q}=p_{u}\frac{\lambda }{\lambda^{Q}}\sum_{j=1}^{n}\frac{p_{j}\eta_{j}}{\eta_{j}^{Q}}. \end{array}$$

Thus, (23) and (24) are fulfilled.

Functions $\exp\left(x\right)$ and $\ln\left(x\right)$ satisfy the assumptions of Proposition 2.3 from [31]. Since they are increasing, for each fuzzy number $\tilde{a}$

(29) $${\left(e^{\tilde{a}}\right)}_{\alpha }=\left[e^{{\tilde{a}}_{\alpha }^{L}},e^{{\tilde{a}}_{\alpha }^{U}}\right]$$

and for each positive fuzzy number $\tilde{b}$

(30) $${\left(\ln\tilde{b}\right)}_{\alpha }=\left[\ln{\tilde{b}}_{\alpha }^{L},\ln{\tilde{b}}_{\alpha }^{U}\right].$$

From the assumptions it follows that $1⊖{\tilde{m}}_{t,T}$, ${\tilde{\xi }}_{i}^{Q}⊕{\tilde{m}}_{t,T}$, ${\tilde{\xi }}_{i}^{Q}⊕1$, i∈[m], ${\tilde{\eta }}_{j}^{Q}⊖{\tilde{m}}_{t,T}$, ${\tilde{\eta }}_{j}^{Q}⊖1$, j∈[n], are positive fuzzy numbers. Straightforward computations employing (29) yield

$$\begin{array}{cc} & {\left({\tilde{m}}_{t,T}\right)}_{\alpha }^{\Xi }=e^{-\left(T-t\right){\tilde{\mu }}_{\alpha }^{\Xi^{\prime }}},\;{\tilde{M}}_{\alpha }^{\Xi }=4{\tilde{\mu }}_{\alpha }^{\Xi },\\ \; & {\left({\tilde{\xi }}_{i}^{Q}\right)}_{\alpha }^{\Xi }={\left({\tilde{\xi }}_{i}\right)}_{\alpha }^{\Xi }+{\bar{\theta }}_{0},\;{\left({\tilde{q}}_{i}^{Q}\right)}_{\alpha }^{\Xi }=\frac{q_{i}{\left({\tilde{\xi }}_{i}\right)}_{\alpha }^{\Xi }/{\left({\tilde{\xi }}_{i}^{Q}\right)}_{\alpha }^{\Xi^{\prime }}}{\sum_{i=1}^{m}q_{i}{\left({\tilde{\xi }}_{i}\right)}_{\alpha }^{\Xi^{\prime }}/{\left({\tilde{\xi }}_{i}^{Q}\right)}_{\alpha }^{\Xi }},\;i\in \left[m\right],\\ \; & {\left({\tilde{\eta }}_{j}^{Q}\right)}_{\alpha }^{\Xi }={\left({\tilde{\eta }}_{j}\right)}_{\alpha }^{\Xi }-{\bar{\theta }}_{0},\;{\left({\tilde{p}}_{j}^{Q}\right)}_{\alpha }^{\Xi }=\frac{p_{j}{\left({\tilde{\eta }}_{j}\right)}_{\alpha }^{\Xi }/{\left({\tilde{\eta }}_{j}^{Q}\right)}_{\alpha }^{\Xi^{\prime }}}{\sum_{j=1}^{n}p_{j}{\left({\tilde{\eta }}_{j}\right)}_{\alpha }^{\Xi^{\prime }}/{\left({\tilde{\eta }}_{j}^{Q}\right)}_{\alpha }^{\Xi }},\;j\in \left[n\right],\\ \; & {\left({\tilde{\lambda }}^{Q}\right)}_{\alpha }^{\Xi }={\tilde{\lambda }}_{\alpha }^{\Xi }\left(q_{d}\sum_{i=1}^{m}q_{i}{\left({\tilde{\xi }}_{i}\right)}_{\alpha }^{\Xi }/{\left({\tilde{\xi }}_{i}^{Q}\right)}_{\alpha }^{\Xi^{\prime }}+p_{u}\sum_{j=1}^{n}p_{j}{\left({\tilde{\eta }}_{j}\right)}_{\alpha }^{\Xi }/{\left({\tilde{\eta }}_{j}^{Q}\right)}_{\alpha }^{\Xi^{\prime }}\right),\\ \; & {\left({\tilde{q}}_{d}^{Q}\right)}_{\alpha }^{\Xi }=q_{d}{\left(\tilde{\lambda }\right)}_{\alpha }^{\Xi }/{\left({\tilde{\lambda }}^{Q}\right)}_{\alpha }^{\Xi^{\prime }}\sum_{i=1}^{m}q_{i}{\left({\tilde{\xi }}_{i}\right)}_{\alpha }^{\Xi }/{\left({\tilde{\xi }}_{i}^{Q}\right)}_{\alpha }^{\Xi^{\prime }},\\ \; & {\left({\tilde{p}}_{u}^{Q}\right)}_{\alpha }^{\Xi }=p_{u}{\left(\tilde{\lambda }\right)}_{\alpha }^{\Xi }/{\left({\tilde{\lambda }}^{Q}\right)}_{\alpha }^{\Xi^{\prime }}\sum_{j=1}^{n}p_{j}{\left({\tilde{\eta }}_{j}\right)}_{\alpha }^{\Xi }/{\left({\tilde{\eta }}_{j}^{Q}\right)}_{\alpha }^{\Xi^{\prime }}. \end{array}$$

and

$$\begin{array}{cc} & {\left({\tilde{F}}^{Q}\left(t,T\right)\right)}_{\alpha }^{\Xi }=\exp{\left[{\tilde{m}}_{t,T}⊗\ln{\tilde{S}}_{t}⊕g\left(T\right)⊖g\left(t\right)⊗{\tilde{m}}_{t,T}⊕{\tilde{\Gamma }}_{t,T}⊘\left(4⊗\tilde{\mu }\right)\right]}_{\alpha }^{\Xi }=\\ & \exp\left[{\left({\tilde{m}}_{t,T}⊗\ln{\tilde{S}}_{t}\right)}_{\alpha }^{\Xi }+g\left(T\right)-{\left(g\left(t\right)⊗{\tilde{m}}_{t,T}\right)}_{\alpha }^{\Xi^{\prime }}+{\left({\tilde{\Gamma }}_{t,T}⊘\tilde{M}\right)}_{\alpha }^{\Xi }\right]=\\ \; & \exp\left[{\left({\tilde{m}}_{t,T}\right)}_{\alpha }^{\Xi }\ln{\left({\tilde{S}}_{t}\right)}_{\alpha }^{\Xi }+g\left(T\right)-{\left(g\left(t\right){⊗}_{int}{\left({\tilde{m}}_{t,T}\right)}_{\alpha }\right)}^{\Xi^{\prime }}+{\left({\left({\tilde{\Gamma }}_{t,T}\right)}_{\alpha }{⊘}_{int}{\tilde{M}}_{\alpha }\right)}^{\Xi }\right]. \end{array}$$

Finally, equality (30) implies (28), which finishes the proof. □

The following corollary is a straightforward consequence of Theorem 2.

**Corollary** **1.** *Let ${\tilde{K}}_{t}$ be the fuzzy forward price of a forward contract calculated at time t. Then, under the above assumptions,*

$${\tilde{K}}_{t}=\frac{1}{N}⊗\overset{N}{\underset{i=1}{⨁}}{\tilde{F}}^{Q}\left(t,T_{i}\right).$$

*Furthermore, for each α∈[0,1] and Ξ∈Σ*

$${\left({\tilde{K}}_{t}\right)}_{\alpha }^{\Xi }=\frac{1}{N}\sum_{i=1}^{N}{\left({\tilde{F}}^{Q}\left(t,T_{i}\right)\right)}_{\alpha }^{\Xi }.$$

## 6. Modified Method of Decision Making in a Fuzzy Environment

In this section, we propose an improved version of a method of investment decision-making, used in our previous papers, i.e., in [13,14], originated from [61], where it was introduced in another context.

Let t∈[0,T]. Denote by ${\hat{K}}_{t}$ the market price of the considered forward price of a forward contract calculated at time *t*. Denote by $V=\left\{B,A,H,R,S\right\}$ the extended set of possible investment decisions: B (to buy; when the contract is significantly undervalued), A (to accumulate; when the contract is undervalued), H (to hold; when the contract is fairly valued), R (to reduce; when the contract is overvalued), and S (to sell; when the contract is significantly overvalued). The advice choice function $\Lambda :R^{2}\mapsto 2^{V}$ takes the form:

$$\begin{array}{cc} \; & B\in \Lambda \left(K_{t},{\hat{K}}_{t}\right)\Leftrightarrow {\hat{K}}_{t}<K_{t};\;A\in \Lambda \left(K_{t},{\hat{K}}_{t}\right)\Leftrightarrow {\hat{K}}_{t}\leq K_{t};\\ \; & H\in \Lambda \left(K_{t},{\hat{K}}_{t}\right)\Leftrightarrow {\hat{K}}_{t}=K_{t};\;R\in \Lambda \left(K_{t},{\hat{K}}_{t}\right)\Leftrightarrow {\hat{K}}_{t}\geq K_{t};\\ \; & S\in \Lambda \left(K_{t},{\hat{K}}_{t}\right)\Leftrightarrow {\hat{K}}_{t}>K_{t}. \end{array}$$

The extended advice choice function $\tilde{\Lambda }:{\left[0,1\right]}^{R}\times R\mapsto {\left[0,1\right]}^{V}$ is obtained with application of the Zadeh extension principle. Let $\tilde{l}$ be the membership function of $\tilde{\Lambda }\left({\tilde{K}}_{t},{\hat{K}}_{t}\right)$. Then,

$$\begin{array}{cc} \; & \tilde{l}\left(B\right)=\min\left(\delta_{{\tilde{K}}_{t}}\left({\hat{K}}_{t}\right),\left(1-\beta_{{\tilde{K}}_{t}}\left({\hat{K}}_{t}\right)\right)\right);\;\tilde{l}\left(A\right)=\delta_{{\tilde{K}}_{t}}\left({\hat{K}}_{t}\right);\\ \; & \tilde{l}\left(H\right)=\min\left(\delta_{{\tilde{K}}_{t}}\left({\hat{K}}_{t}\right),\beta_{{\tilde{K}}_{t}}\left({\hat{K}}_{t}\right)\right);\;\tilde{l}\left(R\right)=\beta_{{\tilde{K}}_{t}}\left({\hat{K}}_{t}\right);\\ \; & \tilde{l}\left(S\right)=\min\left(\beta_{{\tilde{K}}_{t}}\left({\hat{K}}_{t}\right),\left(1-\delta_{{\tilde{K}}_{t}}\left({\hat{K}}_{t}\right)\right)\right) \end{array}$$

and for arbitrary $\hat{x}\in R$:

$$\begin{array}{cc} \beta_{{\tilde{K}}_{t}}\left(\hat{x}\right) & =\sup\left\{\mu_{{\tilde{K}}_{t}}\left(x\right):x\leq \hat{x}\right\}=\left\{\begin{array}{cc} \mu_{{\tilde{K}}_{t}}\left(\hat{x}\right) & \mathrm{for}\;\;{\left({\tilde{K}}_{t}\right)}_{0}^{L}\leq \hat{x}\leq {\left({\tilde{K}}_{t}\right)}_{1}^{L};\\ 1 & \mathrm{otherwise} \end{array}\right.\\ \delta_{{\tilde{K}}_{t}}\left(\hat{x}\right) & =\sup\left\{\mu_{{\tilde{K}}_{t}}\left(x\right):x\geq \hat{x}\right\}=\left\{\begin{array}{cc} \mu_{{\tilde{K}}_{t}}\left(\hat{x}\right) & \mathrm{for}\;\;{\left({\tilde{K}}_{t}\right)}_{1}^{U}\leq \hat{x}\leq {\left({\tilde{K}}_{t}\right)}_{0}^{U};\\ 1 & \mathrm{otherwise}. \end{array}\right. \end{array}$$

One can apply the bisection search (see, e.g., [31]) to receive values of the membership function $\mu_{{\tilde{K}}_{t}}$. The α-level set $\tilde{\Lambda }{\left({\tilde{K}}_{t},{\hat{K}}_{t}\right)}_{\alpha }$ is the extended set of recommendations for a financial analyst.

Recommendations A and R are, respectively, understood as suggestions to prepare for the purchase or sale of the financial instrument. However, in practice, more definite investment decisions are often required. Therefore, we will restrict the set of possible recommendations to the subset $V_{e}\subset V$, where $V_{e}=\left\{B,H,S\right\}$. To this end, we compute the weighted possibilistic mean ${\bar{K}}_{t}=\bar{M}\left(K_{t}\right)$. The final set $D_{\alpha }$ of recommendations for a financial analyst has the form

$$D_{\alpha }=\left(\tilde{\Lambda }{\left({\tilde{K}}_{t},{\hat{K}}_{t}\right)}_{\alpha }\cap V_{e}\right)\cup D_{\alpha }^{\prime },$$

where

$$\begin{array}{c} D_{\alpha }^{\prime }=\left\{\begin{array}{cc} \left\{H\right\} & \mathrm{if}\;\;\tilde{\Lambda }{\left({\tilde{K}}_{t},{\hat{K}}_{t}\right)}_{\alpha }\cap \left\{A,R\right\}=\left\{A,R\right\},{\bar{K}}_{t}={\hat{K}}_{t};\\ \left\{S\right\} & \mathrm{if}\;\;\tilde{\Lambda }{\left({\tilde{K}}_{t},{\hat{K}}_{t}\right)}_{\alpha }\cap \left\{A,R\right\}=\left\{A,R\right\},{\bar{K}}_{t}<{\hat{K}}_{t};\\ \left\{B\right\} & \mathrm{if}\;\;\tilde{\Lambda }{\left({\tilde{K}}_{t},{\hat{K}}_{t}\right)}_{\alpha }\cap \left\{A,R\right\}=\left\{A,R\right\},{\bar{K}}_{t}>{\hat{K}}_{t};\\ \left\{H\right\} & \mathrm{if}\;\;\tilde{\Lambda }{\left({\tilde{K}}_{t},{\hat{K}}_{t}\right)}_{\alpha }\cap \left\{A,R\right\}=\left\{A\right\},{\bar{K}}_{t}\leq {\hat{K}}_{t};\\ \left\{B\right\} & \mathrm{if}\;\;\tilde{\Lambda }{\left({\tilde{K}}_{t},{\hat{K}}_{t}\right)}_{\alpha }\cap \left\{A,R\right\}=\left\{A\right\},{\bar{K}}_{t}>{\hat{K}}_{t};\\ \left\{H\right\} & \mathrm{if}\;\;\tilde{\Lambda }{\left({\tilde{K}}_{t},{\hat{K}}_{t}\right)}_{\alpha }\cap \left\{A,R\right\}=\left\{R\right\},{\bar{K}}_{t}\geq {\hat{K}}_{t};\\ \left\{S\right\} & \mathrm{if}\;\;\tilde{\Lambda }{\left({\tilde{K}}_{t},{\hat{K}}_{t}\right)}_{\alpha }\cap \left\{A,R\right\}=\left\{R\right\},{\bar{K}}_{t}<{\hat{K}}_{t};\\ \emptyset & \mathrm{otherwise}. \end{array}\right. \end{array}$$

## 7. Numerical Examples

### 7.1. Automatized Investment Decision-Making

In this section we will compare on the real-life example the modified investment decision-making method described in Section 6 to the primary one introduced in [13,14], reduced afterwards in [12] to the case of choice out of merely three most significant investment decisions $\left\{B,H,S\right\}$.

Let us assume the following set of the triangular fuzzy numbers (see Table 1, the set of parameters is an extension of those analyzed in [12] in Section 7.1). Usually, ends and modal values of fuzzy numbers are estimated from historical data by traders or supporting them front office in an energy company or a financial institution.

Another market and model parameters which do not undergo fuzzification: $q_{1}=0.35,$ $q_{2}=0.65,$ $p_{1}=0.4,$ $p_{2}=0.6,$ $q_{d}=0.45,$ $p_{u}=0.55,$ t=0, g(0)=4.6, T∈[0.25,0.5], the mean value of the seasonality g(T) over that period is equal to 4.73. Moreover, let us assume that ${\hat{\theta }}_{0}=8,$ ${\bar{\theta }}_{0}=5.$

The resulting modal value of the fuzzy forward price is equal to 137.96, whereas the weighted possibilistic mean ${\bar{K}}_{0}=140.36.$ α is fixed as 0.9. Table 2 illustrates a set of sample market forward prices and a comparison of resulting decisions coming from three proposed investment decision-making methods: a column with the header $\tilde{\Lambda }{\left({\tilde{K}}_{0},{\hat{K}}_{0}\right)}_{0.9}$ shows investment decisions recommended and deeply analyzed in the method proposed in [13,14] (Method 1), the header $\tilde{\Lambda }{\left({\tilde{K}}_{0},{\hat{K}}_{0}\right)}_{0.9}\cap V_{e}$ indicates decisions arising from the method described in [12] (Method 2). Finally, the column marked as $D_{0.9}$ denotes the outcomes from the current’s paper method (Method 3).

The above example encompasses all the possible configurations of investment decisions in Method 1. As can be observed, in this method B is always accompanied by A, S by R and H by A and R (the example is representative because these properties do not depend on the selected α and the magnitudes of ${\hat{K}}_{0}$ and $K_{0}$). Method 2 is reduced compared to Method 1 in order to provide unambiguous indications. However, as can be seen from Table 2, there are some configurations of market data and model parameters when the set of investment decisions is empty for high values of α. The present method, Method 3, fills in this gap, still limiting the final set of recommendations $D_{\alpha }$ to a subset of $\left\{B,H,S\right\}$ basing on the relation between ${\bar{K}}_{0}$ and ${\hat{K}}_{0}$. What is more, the inconclusive set of recommendations $\left\{H,A,R\right\}$ possible in Method 1 is precluded in Method 3. As can be seen in Table 2, when the market price ${\hat{K}}_{0}$ increases from 140 to 141, crossing the weighted possibilistic mean ${\bar{K}}_{0}$, $D_{\alpha }^{\prime }$ changes from $\left\{H\right\}$ to $\left\{S\right\}$ (similar situation would be when ${\bar{K}}_{0}$ was smaller then the modal value of the fuzzy forward price—then the recommendation would change from $\left\{H\right\}$ to $\left\{B\right\}$ when ${\hat{K}}_{0}$ was decreasing and crossing ${\bar{K}}_{0}).$ This is another improvement in comparison to the Method 1, inasmuch as $\left\{R\right\},$ $\left\{R\right\}$ are replaced by unequivocal $\left\{H\right\}$ and $\left\{S\right\}$ for ${\hat{K}}_{0}=140,$ ${\hat{K}}_{0}=141,$ respectively.

The advantage of the weighted possibilistic mean is that it is useful for practical applications—it is a single numeric representation of values present in a fuzzy number. Moreover, the weighting function allows to put emphasis on some subset of the support of a number. Setting f(α)=2α is a natural choice, because the closer an element of a fuzzy number is to the modal value, the bigger the weight. Such a representative of a whole fuzzy number can be easily compared to another numeric indicator, e.g., a market price, which has been used in the formula for the calculation of $D_{\alpha }^{\prime }.$

### 7.2. Price’s α-Level Sets, Membership Function, Sensitivity Analysis

**Figure 1:**
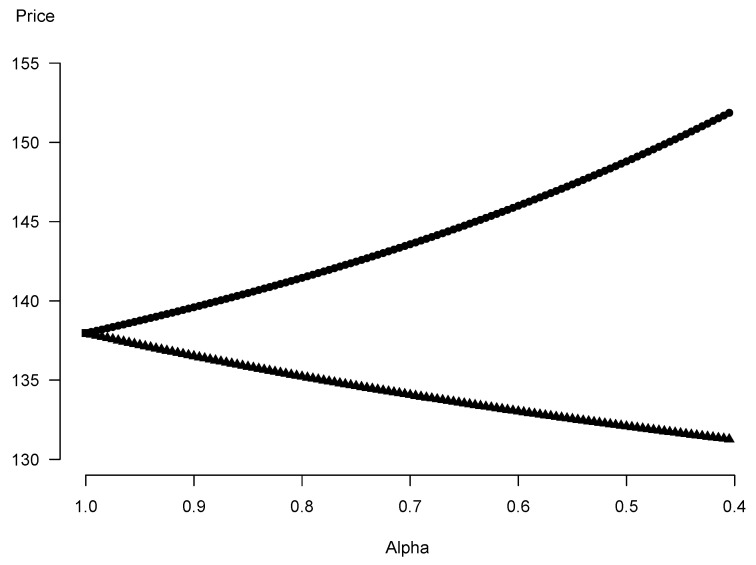
α-Level sets’ ends (circles: right ends, triangles: left ends) of the forward contract’s price depending on the membership degree α.

We continue with the same set of the parameters’ values as in Section 7.1. The relationship between the α-level sets of the fuzzy price of the forward contract and the value of α is shown in Figure 1.

**Figure 2:**
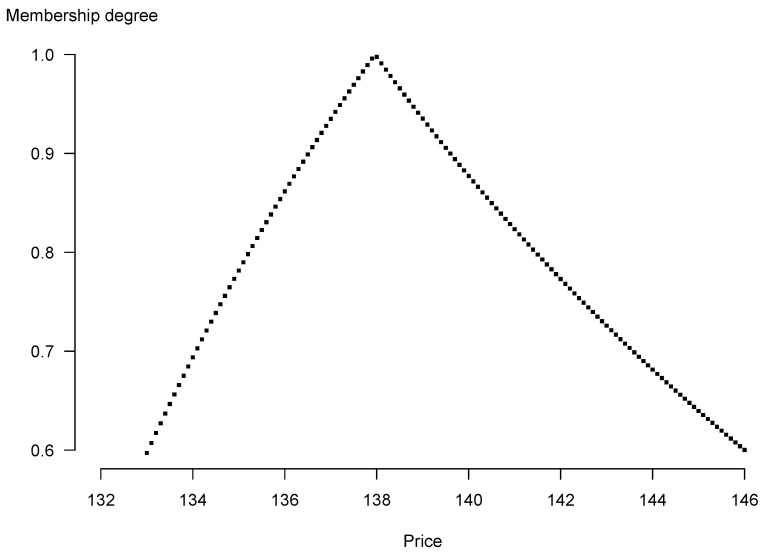
Membership function of the fuzzy forward contract’s price.

Figure 2 illustrates the membership function $\mu_{{\tilde{K}}_{0}}$ of the forward contract’s price. What is remarkable is the triangular shape of the membership function resembling shapes of membership functions of the triangular fuzzy numbers in Table 1. This is a practical proof of stability of the fuzzy pricing method inasmuch as values of the parameters are arbitrarily picked out. From both these plots one can also conclude that the range of values of the right ends of the fuzzy forward price is wider than the range of values of the left ends (the right side of the “triangle” is longer). The possibilistic mean, by its construction, enables to identify and quantify such asymmetry. For our exemplary data the longer right side results in the bigger weighted possibilistic mean (equal to 140.36) than the modal value of the fuzzy price (equal to 137.96). If one selects weighting functions which are convex and therefore take bigger values than the linear f(α)=2α as α becomes closer to 1, the weighted possibilistic mean predominantly mirrors the shape of the membership function near its peak. For the sample data, the difference between legs of the “triangle” diminish when values of the membership function approach 1. This is the reason why for $f\left(\alpha \right)=3\alpha^{2}$ we have ${\bar{K}}_{0}=140$, which is slightly closer to the modal value of the price than picking f(α)=2α.

**Figure 3:**
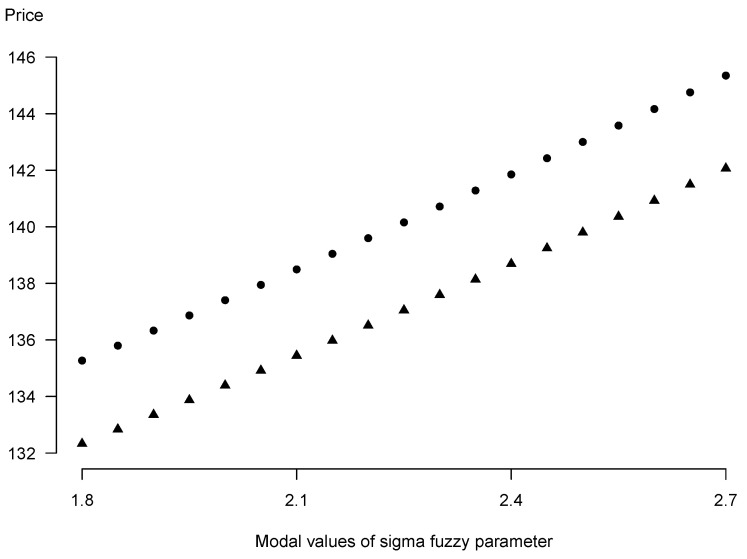
Sensitivity analysis of 0.9-level sets of the fuzzy forward contract’s price to changing triangular number of the parameter σ (circles: right ends of the prices’ intervals, triangles: left ends of the prices’ intervals).

Sensitivity analysis of 0.9-level sets of the fuzzy forward contract’s price with respect to the fuzzified volatility of the underlying $\tilde{\sigma },$ cf. Figure 3, confirms the expected dependence of left and right ends of the forward prices’ intervals on modal values of the parameter (modeled itself as a symmetric triangular number) as an increasing function.

## 8. Conclusions

This paper is devoted to a new stochastic model for the electricity spot prices, which mirrors the specificity of the electrical energy market. We have introduced and discussed the mean-reverting jump-diffusion process of the deseasonalized logarithms of electricity prices with the mixed-exponential probability distribution of jumps, allowing both positive and negative jumps. Using the Esscher transformation for change of probability measure, we have obtained the analytical formulas for the forward contracts’ prices in crisp and fuzzy environments. We have also presented and studied an adjusted method of financial decision-making based on the derived fuzzy pricing formula. The conducted numerical examples have illustrated the theoretical results.

Our future scientific plans involve modeling and valuing the regarded financial derivatives under uncertainty by applying an extended multi-factor model of dynamics of deseasonalized logarithms of electricity prices with stochastic volatility. 

## Figures and Tables

**Table 1:** Exemplary fuzzy parameters estimated from historical data.

$\tilde{S}$	(95, 100, 105)
$\tilde{\mu }$	(110, 115, 120)
$\tilde{\sigma }$	(1.9, 2.2, 2.5)
$\tilde{\lambda }$	(20, 22.5, 25)
$\tilde{\xi_{1}}$	(10, 10.5, 11)
$\tilde{\xi_{2}}$	(14, 14.5, 15)
$\tilde{\eta_{1}}$	(8, 8.5, 9)
$\tilde{\eta_{2}}$	(48, 48.5, 49)

**Table 2:** Automatized decision making for different market prices of the considered forward contract with the modal value of the fuzzy forward price equal to 137.96 and the weighted possibilistic mean equal to 140.36.

${\hat{K}}_{0}$	$\tilde{l}\left(B\right)$	$\tilde{l}\left(A\right)$	$\tilde{l}\left(H\right)$	$\tilde{l}\left(R\right)$	$\tilde{l}\left(S\right)$	$\tilde{\Lambda }{\left({\tilde{K}}_{0},{\hat{K}}_{0}\right)}_{0.9}$	$\tilde{\Lambda }{\left({\tilde{K}}_{0},{\hat{K}}_{0}\right)}_{0.9}\cap V_{e}$	$D_{0.9}$
128.5	0.91	1	0.09	0.09	0	$\left\{B,A\right\}$	$\left\{B\right\}$	$\left\{B\right\}$
135	0.22	1	0.78	0.78	0	$\left\{A\right\}$	*∅*	$\left\{B\right\}$
136.5	0.1	1	0.9	0.9	0	$\left\{H,A,R\right\}$	$\left\{H\right\}$	$\left\{B,H\right\}$
139	0	0.94	0.94	1	0.06	$\left\{H,A,R\right\}$	$\left\{H\right\}$	$\left\{B,H\right\}$
140	0	0.88	0.88	1	0.12	$\left\{R\right\}$	*∅*	$\left\{H\right\}$
141	0	0.82	0.82	1	0.18	$\left\{R\right\}$	*∅*	$\left\{S\right\}$
166	0	0.09	0.09	1	0.91	$\left\{S,R\right\}$	$\left\{S\right\}$	$\left\{S\right\}$

## Data Availability

Not applicable.
